# Musical note onset detection based on a spectral sparsity measure

**DOI:** 10.1186/s13636-021-00214-7

**Published:** 2021-07-28

**Authors:** Mina Mounir, Peter Karsmakers, Toon van Waterschoot

**Affiliations:** 1 KU Leuven, Department of Electrical Engineering (ESAT), STADIUS Center for Dynamical Systems, Signal Processing, and Data Analytics, Kasteelpark Arenberg 10, Leuven, 3001 Belgium; 2 KU Leuven, Department of Computer Science, Declarative Languages and Artificial Intelligence (DTAI), Geel Campus, Kleinhoefstraat 4, Geel, 2440 Belgium

**Keywords:** Note onset detection, Music signal processing, Music signal analysis, Music information retrieval, Sparsity

## Abstract

If music is the language of the universe, musical note onsets may be the syllables for this language. Not only do note onsets define the temporal pattern of a musical piece, but their time-frequency characteristics also contain rich information about the identity of the musical instrument producing the notes. Note onset detection (NOD) is the basic component for many music information retrieval tasks and has attracted significant interest in audio signal processing research. In this paper, we propose an NOD method based on a novel feature coined as Normalized Identification of Note Onset based on Spectral Sparsity (NINOS^2^). The NINOS^2^ feature can be thought of as a spectral sparsity measure, aiming to exploit the difference in spectral sparsity between the different parts of a musical note. This spectral structure is revealed when focusing on low-magnitude spectral components that are traditionally filtered out when computing note onset features. We present an extensive set of NOD simulation results covering a wide range of instruments, playing styles, and mixing options. The proposed algorithm consistently outperforms the baseline Logarithmic Spectral Flux (LSF) feature for the most difficult group of instruments which are the sustained-strings instruments. It also shows better performance for challenging scenarios including polyphonic music and vibrato performances.

## Introduction

Musical note onsets can be simply defined as the start of the notes and hence determine the temporal structure of music, but also play an important role in music color (timbre) perception [1]. This makes note onset detection (NOD) one of the most frequently encountered problems in the arising research fields of machine listening, music processing, and music information retrieval. It can be formulated as an acoustic event detection problem with the signal under processing being a piece of music and the events being the note onsets.

Despite being a long-standing problem, literature does not offer a single but rather different definitions for note onsets. From a music performer’s perspective, it is the time instant at which the performer plays the note. Alternatively, when a note is decomposed into a transient (attack and decay) followed by a steady-state (sustain and release) component [2], an onset can be defined as the start of the note’s attack [3]. Different definitions have led to different (often manual) labeling methods for onset ground truth generation, used in NOD algorithm design and evaluation. While the onset definition of [3] is the key behind the proposed NOD feature, the definition found in [4] considering an onset as the first detectable part of a note in an isolated recording will be considered in this paper for labeling the datasets used for tuning and testing NOD methods based on the proposed and state-of-the-art features.

Note onset detection is increasingly gaining research interest due to its usefulness in many music-related activities and applications: automatic music transcription [5, 6], audio-to-score (audio-to-symbolic representation) alignment [7], music analysis (tempo and beat tracking) [8] [9] and synthesis (enhancement of the attacks of synthesized notes) [10], instrument identification [1], and adaptive audio effects [11], e.g., applying different time stretching for transient and steady-state components of a note. Moreover, onsets are of great importance for music search engines and recommender systems when used as acoustic meta-data about songs [12, Ch. 3].

A general scheme for NOD has been summarized in [3] where the main component is the onset detection function (ODF). The ODF describes the variation of an NOD feature over time and typically represents a highly sub-sampled version of the original music signal from which onsets should be easily detectable as distinctive amplitude peaks [4]. In literature, two main groups of NOD methods can be distinguished: data-driven and non-data-driven methods. Data-driven methods build statistical models for note onsets by employing machine learning methods on a large set of training data. For instance, learning a probabilistic (hidden Markov) model exploiting the rhythmic regularity in music [13], training a neural network (recurrent [14], convolutional [15–18]), or learning dictionaries describing onset/non-onset patterns [7]. These machine learning methods can either solve a classification problem, differentiating onsets from non-onsets, or a regression problem of which the output is then used to estimate a suitable ODF. On the other hand, in non-data-driven methods, the ODF is directly calculated from the analyzed signal or its extracted features. Even though data-driven methods have been shown to slightly outperform non-data-driven methods on the Music Information Retrieval Evaluation eXchange (MIREX) dataset [19], the former need to be trained on large annotated datasets in order to be generically applicable, which is currently impractical as most of the available datasets require manual annotation. Moreover non-data-driven methods are sometimes preferred over data-driven methods as the former allow more easily to find direct relations with the signal’s musical properties compared to the latter.

Non-data-driven NOD methods [2] may operate in the time domain or frequency domain [20] and differ in the type of features extracted from the analyzed signal: magnitude, phase, energy, power spectral density, or time derivatives of these [1]. Many non-data-driven methods exist, see [3] for an early overview: envelope follower, high frequency content (HFC), spectral difference, phase deviation, etc. For a more recent overview, we refer to MIREX [19], where different methods are submitted and evaluated on the same dataset. According to the MIREX results [19], the state-of-the-art non-data-driven method employs the Complex Flux feature [21] which is based on the Super Flux feature [22] taking also phase information into consideration. Both features were proposed to tackle a specific but widely occurring problem (i.e., robustness to vibrato and tremolo effects) and share the basic properties of the Logarithmic Spectral Flux (LSF) feature [20], which in turn is based on the spectral dissimilarity or Spectral Flux (SF) feature proposed earlier in [1] but includes additional pre- and post-processing. This family of features results in an ODF that tracks the temporal evolution of the short-time spectrum based on first-order differences between spectral features in successive signal frames. Several enhancements were proposed to this family, for example adaptive whitening [23], to counter the effect of spectral roll-off. Another related NOD method is the constrained linear reconstruction method [24] which instead of calculating the first-order spectral differences, aims to minimize the spectral difference between the current frame and a group of previous frames and uses the residual as an ODF. These variations add some computational complexity with a slight performance improvement. As all these methods rely on differences between successive frames, the performance of these methods may drop when comparing successive notes sharing a considerable number of harmonics or with repeated notes having insufficient increase in magnitude at their onset. Finally, a more recent approach to NOD using pitch tracking was presented in [25] and performs better with pitched non-percussive instruments.

This paper provides a detailed treatment of a new non-data-driven NOD method based on the NINOS^2^ feature that was proposed earlier by the authors in the context of guitar note onset detection [26]. The NINOS^2^ feature represents a (normalized) sparsity measure of the analyzed signal magnitude spectrum. Even though spectral sparsity implicitly contributes to the operation of the previously discussed non-data-driven methods by differentiating between notes’ transient and steady-state components, it has not been explicitly investigated. Earlier research exploiting signal sparsity in the context of NOD has not resulted in a sparsity measure that can directly be used as an ODF, but has rather focused on the estimation of a sparse signal representation, e.g., by matching pursuit [27] or sparse decomposition [28], from which a suitable ODF is then calculated. In our previous work [26], we focused on guitar notes and chord progressions. Here instead we show and analyze results covering a wider range of instruments and playing styles. Moreover, we provide an in-depth analysis of the NINOS^2^ feature in terms of its sparsity properties, propose two new and improved variants of the feature, compare its computational complexity to the baseline non-data-driven feature, and show its superior performance in challenging NOD scenarios involving polyphonic music, repeated notes, and vibrato effects. Finally, this paper also provides a more detailed explanation of the novel annotation approach introduced in [26], in which semi-synthetic music excerpts are generated and automatically annotated out of isolated musical note recordings.

Having introduced the problem, the related work, and challenges, Section 2 will discuss in further detail the related non-probabilistic methods and their general solution steps. Section 3 provides an in-depth treatment of the NINOS^2^ feature and the resulting novel NOD method. The experimental evaluation is shown in Section 4 comparing NOD results with the NINOS^2^ and LSF features. Finally, Section 5 presents the conclusion and hints for future work.

## Related work

**Fig. 1:**
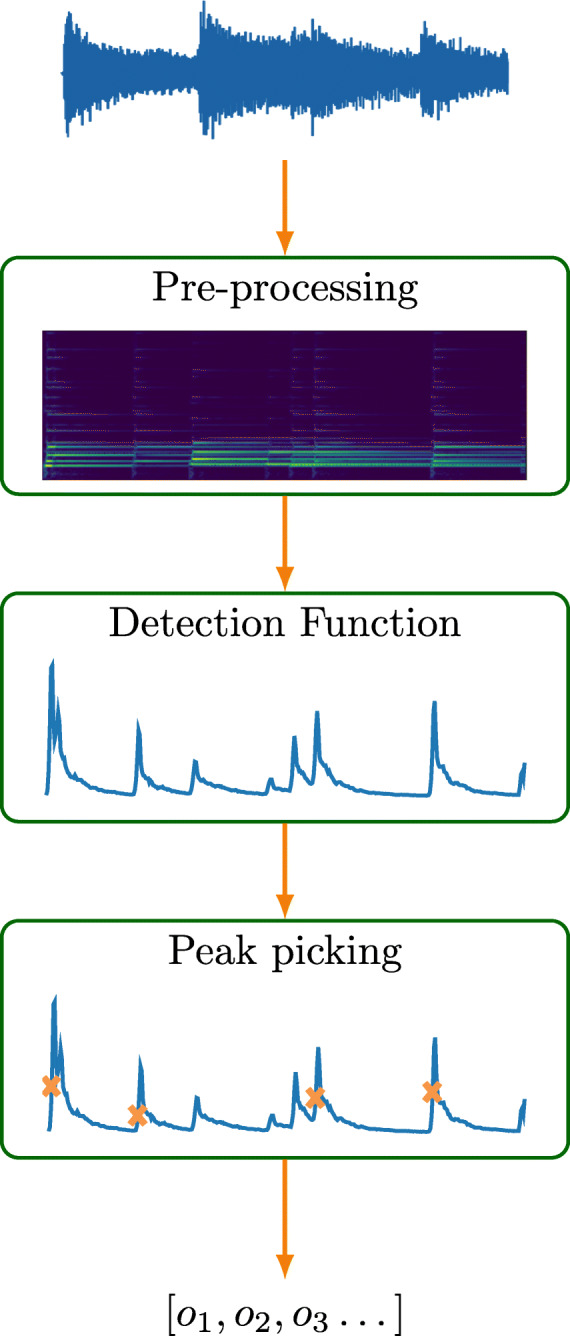
Solution scheme for NOD. An example of the output of the different steps is shown when NOD is applied on an input music excerpt

The majority of non-data-driven NOD methods follow a general scheme that could be divided into three steps, see Fig. 1: pre-processing, reduction, and peak picking [3]. In the pre-processing step, the signal is processed in order to emphasize features related to onsets or to remove noise, which makes the detection easier. The second and most important step is the reduction step in which the ODF, sometimes alternatively named *novelty function*, is computed based on extracted signal features. The resulting ODF is then run through the peak-picking step, in which distinct points marking the estimated onset times are selected from the ODF. This latter step is sometimes referred to as the Onset Selection Function (OSF), and its accuracy highly depends on the quality of the ODF in terms of the presence of noise and artifacts and in terms of the presence and waveform shape of ODF peaks at onset locations [3, 14]. Several OSF approaches have been proposed, either with fixed or adaptive peak picking thresholds, and their impact on the overall onset detection performance has been experimentally assessed [29].

As stated previously, the baseline non-data-driven methods are operating on spectral features of the input signal in order to detect spectral dissimilarities at note onsets. Their respective ODFs are calculated as the first-order difference of the spectral magnitude over successive frames, with some enhancements like adding phase information or adaptive whitening. The SF ODF is defined as follows^1^,

1 $$  \text{SF} \left(n \right) \ = \ \sum_{k=1}^{\frac{N}{2} - 1} \left\lbrace H \left(\lvert X_{k} \left(n\right) \rvert - \lvert X_{k} \left(n-1\right) \rvert \right) \right\rbrace,  $$

where *H* is the half-wave rectification operator keeping track only of positive changes and neglecting the negative ones representing offsets,

2 $$ H\left(x \right) \ = \ \frac{\left(x+ \lvert x \rvert \right)}{2},  $$

*X*_*k*_(*n*) is the *N*-point short-time Fourier transform (STFT) at frame index *n* and frequency *k* of the windowed time-domain input signal *x*(*n*),

3 $$  X_{k}(n) \ = \ \sum_{m=-\frac{N}{2}}^{\frac{N}{2}-1} w(m)x(n+m)e^{-\frac{2j\pi mk}{N}},  $$

and *w*(*m*) is a length-*N* window function. In order to make the ODF invariant to signal scaling, in [30], the first-order difference was applied to the log-magnitude spectrum which is equivalent to the logarithm of the magnitude spectrum ratio between successive frames. In other words, instead of using the STFT magnitude coefficients |*X*_*k*_(*n*)| in (1), the STFT log-magnitude coefficients were used, defined as,

4 $$  Y_{k} \left(n\right) = \log(\lambda \lvert X_{k} \left(n\right) \rvert + 1)\ ,  $$

where *λ* is a compression parameter and a constant value of 1 is added to ensure a positive value for the logarithm. The resulting LSF ODF is defined as

5 $$  \textrm{LSF} \left(n \right) \ = \ \sum_{k=1}^{\frac{N}{2} - 1} \left\lbrace H \left(Y_{k} \left(n\right) - Y_{k} \left(n-1\right) \right) \right\rbrace,  $$

This ODF generally gives better results compared to the SF ODF. We use the LSF ODF for comparison with the proposed method as it is generally considered the baseline non-data-driven method [20]. Optionally, a filterbank according to the Western music scale can be applied as a pre-processing step before the LSF ODF calculation [20].

## Proposed note onset detection method

### Idea: transient vs. steady-state components

A musical note can be considered as a signal consisting of a transient component followed by a steady-state component, where each component can be expressed as a sum of sinusoids. From a sinusoidal modeling perspective, the key difference between the transient and steady-state components of a note is the number of sinusoids needed to represent each. In fact, a transient requires a much larger number of sinusoids than a steady-state component to be accurately approximated. For some percussive instruments, the attack is nearly an impulse signal which conceptually corresponds to the sum of an infinite number of sinusoids. On the other hand, for non-percussive instruments, the transient is stretched over a longer time interval in which it typically also exhibits a broadband behavior which again leads to a representation requiring many sinusoids. This observation also follows from the definition of transients as short time intervals in which the statistical and energy properties of the signal change rapidly [2]. Consequently, the magnitude spectrogram of a musical note shows transients (attacks) that are spectrally less sparse than the subsequent steady-state (tonal) part. Spectral sparsity is considered here along the vertical (frequency) dimension of a magnitude spectrogram and indicates if few or many sinusoids are needed to represent the signal in that particular time frame.

In the following three subsections, we will explain how this idea of spectral sparsity can be realized in the frame of the three-step non-data-driven NOD scheme introduced earlier. At this point, let us start by defining the input to the first step of this scheme. First, the analyzed music signal is divided into overlapping windowed frames and the STFT is computed as in (3). The STFT log-magnitude coefficients *Y*_*k*_(*n*) are then calculated using (4), i.e., in the same manner as for the LSF ODF.

### Pre-processing: coefficients subset

Before calculating the NINOS^2^ ODF, we apply a pre-processing step to maximize the differentiation between the STFT log-magnitude spectra of frames containing onsets and other frames in terms of spectral sparsity. Instead of measuring the spectral sparsity of the entire STFT log-magnitude spectrum of each frame, only a subset of the STFT log-magnitude coefficients will be used. This is motivated as follows.

For the majority of pitched musical instruments, when looking at the magnitude spectrogram of an isolated note recording, it can be observed that the frequency components corresponding to the note’s fundamental frequency and harmonics exhibit high energy during transients followed by a slow energy decrease after the transient. On the other hand, the remaining frequency components, i.e., those not related to the note’s fundamental frequency or harmonics, show a remarkable increase in energy during transients followed by a relatively fast energy decay, i.e., faster than the post-transient energy decay of the harmonics.

**Fig. 2:**
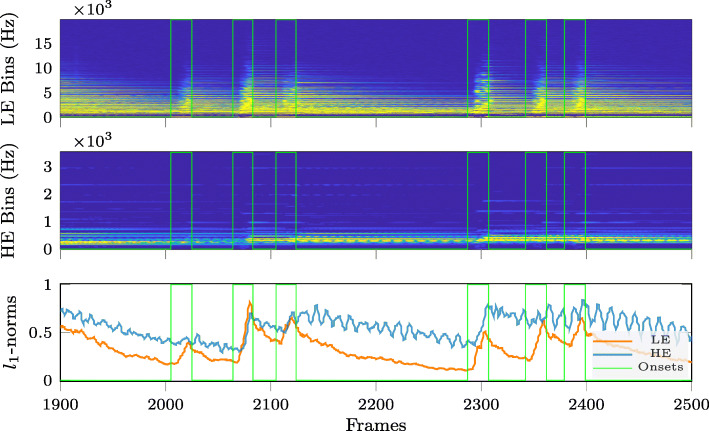
Temporal variation of signal energy per frequency bin and of spectral sparsity for electric guitar (major seventh stopped) excerpt. Note onsets are indicated by vertical lines. (Top and middle) *Low-* and *high-energy* log-magnitude spectrograms. (Bottom) *Low-* and *high-energy* STFT log-magnitude coefficient vector *ℓ*_1_-norm variation

**Fig. 3:**
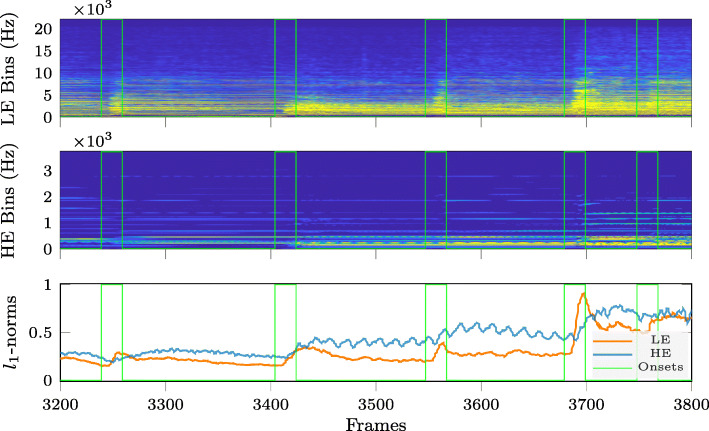
Temporal variation of signal energy per frequency bin and of spectral sparsity for cello (non-vibrato) excerpt. Note onsets are indicated by vertical lines. (Top and middle) *Low-* and *high-energy* log-magnitude spectrograms. (Bottom) *Low-* and *high-energy* STFT log-magnitude coefficient vector *ℓ*_1_-norm variation

**Fig. 4:**
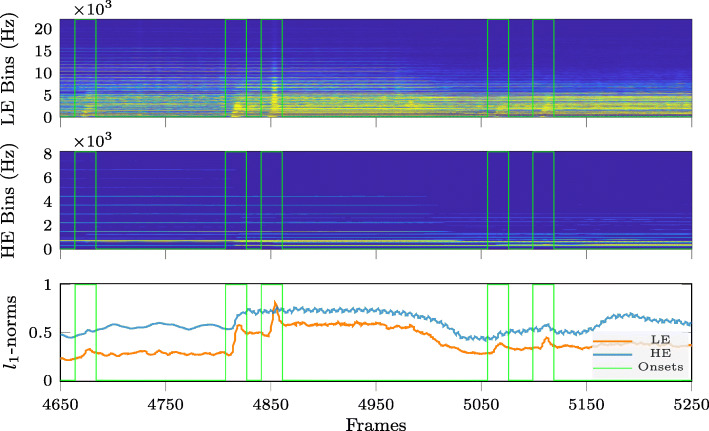
Temporal variation of signal energy per frequency bin and of spectral sparsity for trumpet (Bach) excerpt. Note onsets are indicated by vertical lines. (Top and middle) *Low-* and *high-energy* log-magnitude spectrograms. (Bottom) *Low-* and *high-energy* STFT log-magnitude coefficient vector *ℓ*_1_-norm variation

To illustrate this observation, Figs. 2–4 compare the time variation over 600 signal frames of the energy per frequency bin, after splitting the frequency bins for each time frame into two subsets: one subset containing the frequency bins with low energy (LE Bins, upper subplots in Figs. 2–4) and another subset containing the frequency bins with high energy (HE Bins, middle subplots in Figs. 2–4). This is shown in Figs. 2–4 for three different instruments: electric guitar (Fig. 2), cello (Fig. 3) and trumpet (Fig. 4). It can be clearly observed that the energy variation in the LE Bins exhibits more pronounced peaks in frames containing onsets (marked by the vertical green lines in Figs. 2–4), compared to the energy variation in HE Bins. While the energy in HE Bins does not always increase when reaching an onset, the energy in LE Bins does. Apart from this, it can also be observed that the shape of the energy decay curve after an onset seems to be characteristic for the type of instrument. For instance, the LE Bin energy curves for the electric guitar and the cello do not show an explicit sustain component for their notes while the same curves for the trumpet do show a clear distinction between the transient (attack and decay) and steady-state (sustain and release) components. We can also notice, again from the LE Bin energy curves, that the electric guitar has a faster attack and decay differentiating it from the other two instruments shown here. Finally, the lower subplots for each instrument in Figs. 2–4 show the time variation of the *ℓ*_1_-norm of the vector of STFT log-magnitude coefficients, again separated into the subsets of HE Bins and LE Bins. Indeed, the *ℓ*_1_-norm is one of the simplest sparsity measures [31] and illustrates in these examples that spectral sparsity may be used to detect onsets when applied to the subset of LE Bins.

In summary, removing high-energy STFT log-magnitude coefficients before calculating a spectral-sparsity-based ODF, thus neglecting coefficients corresponding to fundamental frequencies and harmonics, will enhance the discriminative power of the ODF. It is important to emphasize how fundamentally different this pre-processing step is from existing NOD methods. Indeed, pre-processing often emphasizes signal components in HE Bins, considering components in LE Bins to be less relevant for NOD.

The frequency bin subset selection is implemented as follows: the *N*/2−1 STFT log-magnitude coefficients *Y*_*k*_(*n*), *k*=1,…,*N*/2−1 for frame *n* are sorted in ascending order and only the first *J* out of *N*/2−1 coefficients are used afterwards, with

6 $$ J = \left\lfloor \frac{\gamma}{100} \left(\frac{N}{2}-1\right) \right\rfloor,  $$

where ⌊·⌋ denotes the floor function and *γ* represents the percentage of (one-sided) STFT frequency bins to be contained in the LE Bins subset. These *J* coefficients are collected in a length-*J* vector **y**(*n*) and their frequency bin indices are collected in the set $\mathcal {I}_{\text {LE}}(n)$, i.e.,

7 $$ \mathbf{y}(n) = \left[ Y_{k}(n) \right],\ k \in \mathcal{I}_{\text{LE}}(n).  $$

### ODF: spectral sparsity feature

We now provide a detailed derivation of the NINOS^2^ feature and its resulting ODF, starting from the idea of spectral sparsity introduced in Section 3.1. First, after having collected the STFT log-magnitude coefficients of the LE Bins subset during pre-processing, a spectral sparsity feature denoted in [26] as Identifying Note Onsets based on Spectral Sparsity (INOS^2^) is introduced. The INOS^2^ feature is actually an inverse-sparsity measure as to yield large values for non-sparse frames, thus highlighting possible onset locations in time. Considering the INOS^2^ feature per frame *n*, the INOS^2^ ODF is obtained,

8 $$  \Upsilon_{\ell_{2}\ell_{4}}(n) = \frac{\lVert \mathbf{y}(n) \rVert^{2}_{2} }{\lVert \mathbf{y}(n) \rVert_{4}} = \frac{\sum_{k \in \mathcal{I}_{\text{LE}}(n)} Y_{k}^{2}(n)}{\left(\sum_{k \in \mathcal{I}_{\text{LE}}(n)} Y_{k}^{4}(n)\right)^{\frac{1}{4}}}.  $$

We will first argue why the INOS^2^ feature can indeed be interpreted as an inverse sparsity measure, before considering how it could be further improved for the purpose of NOD. Even though sparsity is usually defined as the number of non-zero elements in a vector, which corresponds to its *ℓ*_0_-pseudonorm, there is no universal consensus for defining and measuring sparsity [31]. When measuring sparsity of signal vectors that contain noise or other artifacts, the *ℓ*_0_-pseudonorm becomes useless since it cannot discriminate between small (“near-to-zero”) and large (“far-from-zero”) elements in the vector. Two basic conditions that should be satisfied by any sparsity measure have been stated in [31]: the most sparse signal is the one having all its energy concentrated in one sample while the least sparse is the one having its energy uniformly distributed over all its samples. For example, according to this definition, a measure *S* that satisfies the following inequalities for vectors of equal length would indeed be considered a sparsity measure,

9 $$\begin{array}{*{20}l} S_{max} &= S([0,0,0,0,1])> \dots >S([0,0,1,1,1]) \\ & > \dots >S([1,1,1,1,1]) = S_{min}. \end{array} $$

where *S* is a sparsity measure defined for vectors of equal length. Using this dummy example, it can be verified that the INOS^2^ feature in (8) is indeed an inverse sparsity measure. However, this observation only holds if the vectors in comparison have similar energies. For instance, according to the INOS^2^ feature, the vector *v*=[0,0,100,100,100] is less sparse than the vector [1,1,1,1,1], which clearly contradicts with the basic conditions for a sparsity measure given above. The explanation for this behavior is that the INOS^2^ feature is in fact a joint energy and inverse sparsity measure. This becomes clear when rewriting (8) as follows,

10 $$  \Upsilon_{\ell_{2}\ell_{4}}(n) = \lVert \mathbf{y}(n) \rVert_{2} \cdot \frac{\lVert \mathbf{y}(n) \rVert_{2} }{\lVert \mathbf{y}(n) \rVert_{4}}.  $$

**Fig. 5:**
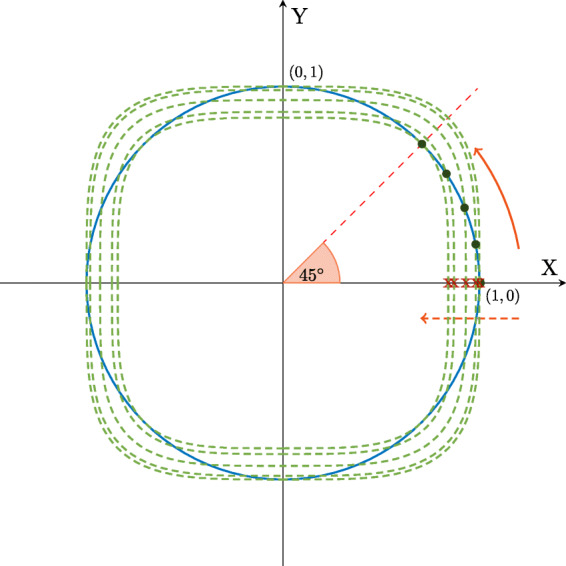
2-D unit-ball illustration of the relation between the *ℓ*_2_-norm and *ℓ*_4_-norm

The first factor ∥**y**(*n*)∥_2_ is the vector *ℓ*_2_-norm which directly relates to the signal frame’s energy. As onsets are usually accompanied by an increase in energy, see, e.g., Figs. 2–4, this property of the INOS^2^ feature is actually desirable for NOD. In fact, the use of a pure energy measure was proposed as one of the earliest features for NOD, resulting in an ODF known as the envelope follower [1]. The second factor in (10), i.e., the ratio between the signal frame’s *ℓ*_2_-norm and *ℓ*_4_-norm, can be understood to measure inverse sparsity by applying the unit-ball concept [2] as shown in Fig. 5.

The figure shows a 2-D coordinate system where each point is representing a vector $\mathbf {v} \in \mathbb {R}^{2}$. Focusing on the first quadrant, it can be noticed that points near the *X* or *Y* axis, represent sparse vectors and inversely, while moving away from the axes towards the 45^∘^ line (e.g., moving away from the *X* axis in the direction of the (solid) red arrow), vectors get less sparse. By applying the basic conditions for a sparsity measure given above, the most sparse vectors correspond to points lying on the axes, e.g., [0,1] and [1,0], while points lying on the ±45^∘^ lines correspond to the least sparse vectors, e.g. $[ \sqrt {0.5},\sqrt {0.5}]$ lying on the unit circle. Now, let us consider the relation between *ℓ*_*p*_-norms for different *p* in this 2-D coordinate system. The unit circle represents the *ℓ*_2_-norm unit ball, i.e., the set of points representing vectors having an *ℓ*_2_-norm equal to 1. For each of the points on the *ℓ*_2_-norm unit ball, we can graphically evaluate the *ℓ*_4_-norm by looking at the *ℓ*_4_-norm unit ball and its scaled versions each containing one of the points. We observe that the scaled *ℓ*_4_-norm ball gets smaller, shrinking along the dashed red arrow, while considering a point moving along the (solid) red arrow. This means the ratio of the *ℓ*_2_-norm and the *ℓ*_4_-norm increases as the vector becomes less sparse.

Apart from the two basic conditions discussed above, a number of additional desirable properties of a sparsity measure have been proposed in [31]. These properties and their interpretation for a sparsity-based NOD feature are briefly summarized here:

- *Robin Hood*: Taking from the richest (highest-energy signal samples) and giving to the poor (lowest-energy signal samples) decreases the sparsity of a signal. Considering the analogy with spectral sparsity in the NOD context, the transient component of a note represents a scenario with many poor where energy is fairly distributed, after which the energy shifts to the harmonics in the steady-state component which represents a scenario with few rich.
- *Scaling*: Sparsity is independent of signal scaling, e.g., *S*([0,0,1,1,1])=*S*([0,0,100,100,100]). This is an important property in terms of decoupling an NOD feature into an energy measure and sparsity measure, as discussed above when factorizing the INOS^2^ feature as in (10).
- *Rising Tide*: Adding a constant value to each of the signal samples decreases sparsity.
- *Cloning*: Sparsity measures should preferably assign the same value to cloned signals, i.e., $S([0, 1]) = S([0, 0, 1, 1]) = S([0, 0, 0, 1, 1, 1] = \dots)$. As will be discussed below, this property is relevant to reduce the amount of computations needed for the NOD feature calculation and to make the resulting ODF insensitive to the use of frames with different frame lengths.
- *Bill Gates*: As an individual gets richer, sparsity increases. In the context of NOD, as the steady-state component of a note shows a more pronounced tonal behavior, i.e., the more its energy is concentrated in a few frequency components, it will be easier to discriminate the transient from the steady-state component using a spectral sparsity
- *Babies*: Appending zeros to a signal increases its sparsity.

The ratio of a signal vector’s *ℓ*_4_-norm and *ℓ*_2_-norm, i.e., the inverse of the second factor of the INOS^2^ feature in (10), can be understood to satisfy all of the above properties except for the cloning property. In other words, this ratio is sensitive to changes in the vector length, i.e., *J* in the context of the INOS^2^ feature. This is an undesirable property for an NOD feature for several reasons. Firstly, it makes the choice of a detection threshold and peak-picking parameters dependent on the frame size *N* and pre-processing parameter *γ*. Secondly, it makes the feature incompatible with a processing strategy in which successive signal frames may have different lengths to achieve detections with different time resolutions (which is however outside the scope of this paper). Finally, it would yield different feature values for the cases that the full frequency grid or only the positive frequency grid is used in the feature calculation, as briefly touched upon earlier ^[1]^.

For these reasons, the INOS^2^ feature can be improved by normalizing the inverse-sparsity factor in (10) such that it yields a value ∈[0,1] independently of the length *J* of the vector **y**(*n*). Let us denote the inverse-sparsity factor in (10) as $\mathcal {S}(n)= \lVert \mathbf {y}(n) \rVert _{2} / \lVert \mathbf {y}(n) \rVert _{4}$ and its (theoretically achievable) minimum and maximum value as $\mathcal {S}_{\min }$ and $\mathcal {S}_{\max }$, respectively. A normalized version of $\mathcal {S}(n)$, i.e., $\mathcal {\bar S}(n) \in [0,1]$ can then be obtained as follows,

11 $$ \mathcal{\bar S}(n) = \frac{\mathcal{S}(n) - \mathcal{S}_{\min}}{\mathcal{S}_{\max}-\mathcal{S}_{\min}}.  $$

The values $\mathcal {S}_{\min }$ and $\mathcal {S}_{\max }$ can be found by considering two extreme cases of an arbitrarily scaled length-*J* vector: the sparsest possible vector $\left [ a,0,0,\dots,0\right ]$ having $\mathcal {S}(n) = 1 \triangleq \mathcal {S}_{\min }$ and the least sparse vector $\left [ a,a,a,\dots,a\right ]$ having $\mathcal {S}(n) = \sqrt [\leftroot {-2}\uproot {3}4]{J} \triangleq \mathcal {S}_{\max }$. By substituting these values in (11) we obtain that

12 $$ \mathcal{\bar S}(n) = \frac{\mathcal{S}(n) -1}{\sqrt[\leftroot{-2}\uproot{3}4]{J} -1} = \frac{\frac{\lVert \mathbf{y}(n) \rVert_{2}}{\lVert \mathbf{y}(n) \rVert_{4}} -1}{\sqrt[\leftroot{-2}\uproot{3}4]{J} -1}.  $$

Combining this normalized inverse-sparsity factor with the energy factor in (10) finally results in the normalized version of the INOS^2^ feature denoted as NINOS^2^. Considering the NINOS^2^ feature per frame *n*, the NINOS^2^ ODF is obtained,

13 $$  \aleph_{\ell_{2}\ell_{4}}(n) \ = \frac{\lVert \mathbf{y}(n) \rVert_{2}}{\sqrt[\leftroot{-2}\uproot{3}4]{J} -1} \left(\frac{\lVert \mathbf{y}(n) \rVert_{2}}{\lVert \mathbf{y}(n) \rVert_{4}} -1 \right).  $$

This definition of the NINOS^2^ ODF slightly differs from the original definition in [26] because of the different normalization procedure. The two definitions deviate in particular for relatively small values of *J*, and only the definition proposed here in (13) guarantees that the inverse-sparsity factor $\mathcal {\bar S}(n) \in [0,1]$.

Finally, in this paper, we also propose a new sparsity-based NOD feature, starting from the observation that the above unit-ball demonstration can be applied to any ratio of norms, ∥**y**(*n*)∥_*p*_/∥**y**(*n*)∥_*q*_, where *p*<*q*. A particularly interesting choice is *p*=1,*q*=2, since in this case the energy factor ∥**y**(*n*)∥_2_ cancels out with the denominator of the inverse sparsity factor ∥**y**(*n*)∥_1_/∥**y**(*n*)∥_2_, and the joint energy and inverse sparsity measure reduces to the *ℓ*_1_-norm, i.e., (compare to (10)),

14 $$  \Upsilon_{\ell_{1}}(n) = \lVert \mathbf{y}(n) \rVert_{2} \cdot \frac{\lVert \mathbf{y}(n) \rVert_{1} }{\lVert \mathbf{y}(n) \rVert_{2}} = \lVert \mathbf{y}(n) \rVert_{1}.  $$

When applying the same normalization strategy as above, the *ℓ*_1_-norm version of the NINOS^2^ ODF is readily obtained,

15 $$  \aleph_{\ell_{1}}(n) \ = \frac{\lVert \mathbf{y}(n) \rVert_{2}}{\sqrt{J} -1} \left(\frac{\lVert \mathbf{y}(n) \rVert_{1}}{\lVert \mathbf{y}(n) \rVert_{2}} -1 \right).  $$

However, by comparing the expressions in (14) and (15), it is clear that the non-normalized expression is much cheaper to compute due to the cancelation of the *ℓ*_2_-norm in (14). Therefore, we will further consider the INOS^2^ (*ℓ*_1_) ODF in (14) as a computationally appealing alternative to the NINOS^2^ (*ℓ*_2_*ℓ*_4_) ODF in (13).

### Peak-picking

To allow for a fair comparison of the proposed ODF with the state of the art, the same peak-picking procedure as used with the baseline non-data-driven NOD method in [21] is adopted here. According to this procedure (shown here for the NINOS^2^ (*ℓ*_2_*ℓ*_4_) ODF and similar for the other ODFs), an onset is detected in the *n*th signal frame if *all* of the following conditions are satisfied, (1) $\aleph (n) = \max _{l} \ \aleph (n+l), \quad \text {with} \quad l = -\alpha,\dots, +\beta $, (2) $\aleph (n) \geq \ \frac {1}{a+b+1} \sum _{l=-a}^{+b} \aleph (n+l) + \delta $, (3) *n*−*p*>*Θ*,

where *α*, *β*, *a*, *b*, and *Θ* are the peak-picking parameters defined using the following terminology, *before maximum*, *after maximum*, *before average*, *after average*, and *combination width*, all counted in frame units, and *p* is the frame index of the previously detected onset. The interpretation of the above conditions is that (1) an onset should correspond to the highest ODF amplitude in the neighborhood of the frame index *n*, (2) an onset’s ODF amplitude should be an amplitude offset *δ* above its neighborhood average ODF amplitude, and (3) an onset should be *Θ* frames apart from the closest preceding onset.

While the peak-picking parameter values are kept the same as in [21], the combination width *Θ* is set equal to the detection window length which is the maximum number of frames in which a single ground-truth onset could occur. This value depends on the frame overlap and is calculated using the following relations:

16 $$\begin{array}{*{20}l} h &= \lfloor (1-q) N \rceil, \end{array} $$

17 $$\begin{array}{*{20}l} r &= f_{s} / h, \end{array} $$

18 $$\begin{array}{*{20}l} \Theta &= \lceil r N / f_{s} \rceil, \end{array} $$

where ⌊·⌉ and ⌈·⌉ denote the nearest integer and ceiling functions, *h* is the frame hop size in samples, *q*∈[0,1] is the frame overlap factor, *N* is the frame size in samples, *r* is the frame rate, *f*_*s*_ is the sampling frequency, and *Θ* as defined above is the number of frames to be skipped after one onset detection before aiming to detect a new onset. It is advisable to choose the value of *Θ* greater than the value given in (18) in case of instruments with very long attacks.

### Onset detection parameters

For a complete understanding of the new non-data-driven NOD method based on the (N)INOS^2^ feature and of the performance measures explained further, the most important parameters impacting the method’s NOD performance are summarized here:

- *Processing frame size* (*N*): It should be larger than a single period of the signal [1] yet small enough to capture transients.
- *Detection resolution*: It depends on the frame rate *r* which is inversely proportional to the hop size *h*.
- *Processing mode*: The detection algorithm could be run in either *offline* or *online* mode. In the latter, the peak-picking parameters *β* and *b* are set to zero.
- *Evaluation window*: It is used to increase the detection window—implementing an onset ground-truth—to handle the lack of precision inherent in the onset annotation process, i.e., onsets may occur slightly before or after the annotated ground-truth onset.

## Experiment setup and evaluation

### Dataset

As mentioned earlier, the majority of NOD results found in literature are obtained by testing on manually annotated datasets. A manual annotation process consists in asking music experts to listen to a number of music excerpts and to inspect their waveforms and spectrograms in order to find the onset times. The final annotation is obtained by averaging the different experts’ onset times. Some NOD results reported in literature are instead obtained by testing on automatically annotated datasets [26, 32]. Various ways of achieving automatic annotation have been proposed [33, 34], e.g., by using a musical instrument equipped with electromechanical sensors or by synthesizing music signals starting from isolated note recordings. The choice to test NOD methods with either manually or automatically annotated datasets depends on several factors. The main advantage of manual annotation over automatic annotation is that manually annotated onsets represent perceived note onsets rather than onsets based on some non-perceptual detection threshold. This advantage however only holds to some extent, as manual annotation is often based on audiovisual rather than purely auditive inspection of an audio file. Arguments in favor of automatic annotation are its ease of deployment in generating large annotated datasets, as manual annotation is time-consuming and labor-intensive, and its potential to yield objective and systematic results independent of manual annotation errors or subjective differences across human annotators.

With these arguments in mind, in this paper, we use automatically-annotated semi-synthetic datasets. To this end, a Matlab tool called “Mix Notes” has been developed by the authors to generate music excerpts from isolated note recordings together with their respective ground-thruth onset annotation^2^. The tool loads isolated note and/or chord recordings from a database and then automatically determines the ground-truth note onsets (and offsets) based on a short-term energy measure. This can indeed be done easily and accurately when notes are individually recorded and avoids the dependency of the annotation on the musical note context. Here, we used a simple energy measure: the onset is chosen to be the earliest point in time *n*_*o*_ at which the absolute value of the signal amplitude |*x*(*n*_*o*_)| satisfies that

19 $$ x(n_{o}) \geq \frac{\rho}{100} \cdot \max_{n} \ |x(n)|, \quad 0 <\rho \ll 100,  $$

where *ρ* is a percentage of the note’s highest amplitude and should define the amplitude threshold of a just audible sound. The tool then generates a melody by automatically mixing the selected note recordings while imposing a specified minimum time spacing between successive onsets. An annotation file for the generated melody is automatically created using the isolated note annotations with the timing information from the mixing process.

For the evaluation of the NOD methods, in this paper, we have created an automatically annotated semi-synthetic dataset using the MixNotes tool with isolated note recordings of various instruments and playing styles, starting from the recordings in the McGill University Master Samples (MUMS) library [35]. Before annotating the isolated note recordings and generating the semi-synthetic music excerpts, all files in the MUMS library were checked for compatibility with the NOD problem. The files were manually checked and retained only when containing just one instance of the respective note. In summary, the *harp* was excluded as its recordings were mostly composed of many notes, e.g., a glissando recording, in one file. All the percussions’ rolling recordings and the library folder “Percussions patterns and dance” were excluded for the same reason. Moreover, the *skins* and *metals* percussions folders were each divided into 3 folders—respecting the alphabetical order of file names—as these contain many more note recordings than other instruments. This resulted in 138 folders containing different instruments with different playing styles. For a more structured performance evaluation, the folders were grouped using a similar grouping as used in the MIREX NOD dataset [19]. The grouping scheme is shown in Table 1 using similar naming as the MUMS folder names. The *bowed vibraphone* instrument is excluded from the grouping as it does not fit in any of the groups.

**Table 1:** Instrument grouping

*Pianos*	
Harpsichord	8 Stop, 8_4 Stops, Buff Stop.
Piano	Concert Hall Steinway Soft, Hamburg Steinway Loud,
	Harmonics, Mpp Loud, Mpp Medium, Mpp Soft, Right Pedal Vol9, Steinway Plucked.
*Bars and bells*	
Marimba	Grand Symphonic, Soft Mallet.
Steel drum	Loud, Soft.
Vibraphone	Hard Mallet, Soft Mallet.
Others	Celesta, Crotales, Tubular Bells, Xylophone.
*Brass*	
French horn	Normal, Muted.
Trombone	Pedal Notes, Tenor, Tenor Muted, Alto, Bass.
Trumpet	In C, In C_Harmon Mute, Bach, Bucket_Loud, Bucket_Soft, Cup_Loud, Hard Attack.
Others	Accordion Treble Notes, Cornet, Tuba.
*Winds*	
Sax	Alto, Alto Growls, Alto Screams, Baritone, Bass, Soprano, Tenor, Tenor Growls, Tenor Subtones.
Clarinet	Bflat, Bass, Contrabass, Eflat.
Flute	Alto_Non Vib, Alto_Vib, Bass_Flutter, Bass_Non Vib, Bass_Vib, Flutter, Non Vib, Vibrato.
Pan flute	High, Low, Medium.
Piccolo	Flutter, Non Vib, Vibrato.
Others	Bassoon, Contrabassoon, English Horn, Oboe.
*Plucked strings*	
Acoustic bass	Amplified, Pizz, Plucked, Slides.
Acoustic guitar	Normal, Normal(2), Pizzicato, Sul Ponticello, Sul Tasto.
Electric guitar	Normal, Harmonics, Stereo Chorus.
Others	Archluteoud, Cello Pizzicato, Renaissance 8 Course Lute, Viola Pizzicato, Violin Pizzicato, Vln Ensemble_Pizzicato-Wet.
*Sustained strings*	
Acoustic bass	Bowed_Vibrato, Harmonics, Martele, Muted.
Viol	Bass, Tenor, Treble.
Cello	Normal, Artificial Harmonics, Martele, Muted_Vibrato, Natural Harmonics, Non-Vibrato.
Viola	Artificial Harmonics, Martele, Muted, Natural Harmonics, Non-Vibrato, Vibrato.
Violin	1 Non Vibrato, 2 Non Vibrato, 3 Non Vibrato, Harmonics_Artificial, Harmonics_Natural, Martele, Muted_Vibrato, Vibrato.
Vln Ensemble	Dry-Bright, Harmonics-Wet, Martele-Wet, Soft Attack-Wet.
*Percussions*	
Acoustic guitar tapping, metals-1/2/3, skins-1/2/3, tympani.	
*Polypitched*	
Electric guitar	Dominant Ninths, Dominant Sevenths, Elevenths, Flat 7 Sharp 9, Major Seventh, Major Seventh Stopped, Major Sixths,
	Minor Sevenths, Ninths, Fifths.
Others	Accordion Chords.

The proposed and baseline non-data-driven NOD methods are evaluated on these 138 folders covering a large number of instruments (electric, acoustic, wind, brass, strings,percussions, etc.) and playing styles (notes, chords, pizzicato, sul ponticello, bowed, screams, flutter, etc.). For each instrument and playing style, i.e., each separate folder, three test melodies are generated, each consisting of a sequence of 50 randomly selected notes. The first melody is used for tuning the NOD algorithm parameters whereas the other two are used for performance evaluation. The time spacing between two notes in each generated melody is randomly chosen from a uniform distribution on the interval [5250,44100] samples which, at a sampling frequency *f*_*s*_=44.1 kHz, corresponds to [0.12,1] s. These values were chosen to limit the onsets per minute to 500, which is slightly above the maximum onsets per minute of an average pop song with a tempo of 120 beats per minute and 4 notes per beat, i.e., 16th notes.

The above dataset generation procedure is repeated four times to produce four datasets with distinct properties relevant to NOD performance evaluation:

- *M* dataset: monophonic mixing.
- *MR8* dataset: monophonic mixing with forced repeated notes where every note is repeated 8 times.
- *P* dataset: polyphonic mixing.
- *PR8* dataset: polyphonic mixing with forced repeated notes where every note is repeated 8 times.

The *M* and *P* datasets (and equivalently the *MR8* and *PR8* datasets) are composed of the same note sequences with the same onset times and time spacings. However, in the *M* and *MR8* datasets, an exponential amplitude decay is applied to the sustain part of the notes, such that each note has faded out before the next note starts, effectively forcing a monophonic music excerpt. In the *P* and *PR8* datasets, successive notes are allowed to overlap in time. The use of repeated notes in *MR8* and *PR8* is mainly to assess the algorithms’ performance in detecting onsets of successive notes that are sharing a considerable number of harmonics with insufficient increase in magnitude at their onset. These latter datasets are expected to be challenging for the baseline algorithm based on spectral differences.

### Performance measures

An important issue when assessing detection performance is to decide how true positives and negatives are counted. Here, we adopt the same approach as used in the evaluation of the state-of-the-art method [22], where two onsets detected within one detection window are counted as one true and one false positive, and a detected onset can count only for one detection window.

The most common way to compare NOD methods is by evaluating their relative F1-scores. The F1-score is defined as the harmonic mean of the precision (*P*) and the recall (*R*) given by:

20 $$ F1 = \frac{2 P R}{P+R},  $$

with the precision being the ratio of correctly detected onsets (“true positives”) to the total number of onsets under test, and the recall comparing the number of correctly detected onsets to the total number of points detected as onsets.

Since a true positive could occur anywhere within the detection window, a new measure is developed to determine how large the detection window should be in order to achieve the reported F1-score. For each true positive in the music excerpt under test, the time difference of the ground-truth onset frame to the first frame that is covered by the detection window is saved, and then the *Detections standard deviation* *σ*_*d*_, i.e., the standard deviation of the time difference of the ground-truth onset to the start of the detection window, is computed for each music excerpt used in the evaluation. When *σ*_*d*_ is small, the algorithm will be detecting onsets in the same relative position to the start of the detection window. This is an important measure that reflects how well the algorithm detects the different onset times relative to each other.

### Computational complexity and memory usage

The running time of a non-data-driven NOD method is comparable when using the LSF or NINOS^2^ ODFs and considerably shorter when using the INOS^2^ (*ℓ*_1_) ODF. Whereas the computation of the NINOS^2^ feature is slightly more expensive than the LSF feature, mainly due to the required pre-processing, the opposite is true for the memory usage as the NINOS^2^ feature does not require any information from the previous frame. Here, we compare the type and number of operations and the amount of memory needed for the computation of the three features, omitting the calculation of the STFT log-magnitude coefficients and the peak picking which are shared by all methods. Note that for all features we only use half minus one, i.e., $\frac {N}{2}-1$, of the FFT coefficients.

The number of operations and memory positions needed for the LSF feature calculation is

$$\begin{array}{*{20}l} \mathcal{C}_{\textrm{LSF}} &= \left(\frac{N}{2}-1\right) Sub + \left(\frac{N}{2}-2\right) Add \\ & + \left(\frac{N}{2}-1\right) Comp\\ \mathcal{M}_{\textrm{LSF}} &= N-2 \end{array} $$

with *Sub*, *Add*, and *Comp* representing scalar subtractions, additions, and comparisons respectively. Here, the comparisons are needed for the half-wave rectification. The number of operations and memory positions needed for the (N)INOS^2^ feature calculation is (with $J < \frac {N}{2}-1$, see (6)),

$$\begin{array}{*{20}l} \mathcal{C}_{{\text{NINOS}_{2}}(\ell_{2}\ell_{4})} &= (2J+1) Mult + 2(J-1) Add + 3\ Sqrt\\ & + 1\ Sub + 1\ Div \\ & + \left(\frac{N}{2}-1\right) \log\left(\frac{N}{2}-1\right) Comp\\ \mathcal{C}_{{\textrm{INOS}_{2}}(\ell_{1})} &= (J-1) Add \\ & + \left(\frac{N}{2}-1\right) \log\left(\frac{N}{2}-1\right) Comp\\ \mathcal{M}_{{\textrm{(N)INOS}_{2}}} &= \frac{N}{2}-1 \end{array} $$

with *Mult*, *Sqrt*, and *Div* representing scalar multiplications, square roots, and divisions, respectively. Here, the comparisons are needed for the frequency-bin sorting in the pre-processing. Note that the computation of the *ℓ*_1_-norm in (14) does not require the calculation of absolute values since the elements of **y**(*n*) are positive by construction, see (4).

### Parameter tuning

The non-data-driven NOD methods presented here are dependent on a number of parameters: input preparation, pre-processing, reduction, and peak picking parameters. Finding the optimal combination of parameter values is not an easy task. In this paper, the different parameters are tuned in a sequential manner; in other words, the parameters are selected one after another. We will now summarize how the different parameters have been tuned, making use of the tuning dataset introduced in Section 4.1.

The input test melodies are sampled with a sampling frequency *f*_*s*_=44.1 kHz. Each melody is divided into overlapping frames of *N*=2048 samples (46.4 ms). This frame size value was chosen by looking at the resulting spectrograms for three different frame sizes: 1024, 2048, and 4096. These are all powers of 2 to benefit from using the fast Fourier transform algorithm. By repeating this for various instruments, *N*=2048 was found the best trade-off between frequency and time resolution in (visually) emphasizing onsets in the spectrogram. By maximizing the F1-score over the tuning dataset, we found the Hanning window offering a better performance than the Hamming window, and the Discrete Fourier Transform outperformed the Discrete Cosine Transform in computing the STFT coefficients. The compression parameter *λ* was set to 1 as in the Madmom implementation [36]. A frame overlap of 90%, i.e., *q*=0.9, is used similarly to the baseline method, resulting in a frame rate of *r*=215 frames per second or a 4.6 ms detection resolution which is better than the temporal hearing resolution (≈10 ms). The evaluation window is set to ±25 ms around the ground-truth onset.

Due to the fact that the NINOS^2^ feature satisfies the cloning property (D4 in [31]), using only 50% of the STFT coefficients will give the same results. Note that this does not hold for the non-normalized INOS^2^ feature. The percentage *γ* of STFT frequency bins used to compute the (N)INOS^2^ feature is set to value *γ*=95.5 %, which was found to maximize the F1-score over the tuning dataset for *γ* values lying between 90 and 99.

The peak-picking parameters *α*=30 ms and *a*=100 ms are kept as in [21] as these values resulted in better F1-scores for all the algorithms compared to the values found in [36]. The parameters *β* and *b* are set to zero to allow the NOD methods to operate online. The parameter *Θ* is set equal to the detection window length as stated earlier. Finally, the amplitude offset *δ*—sometimes referred to as the detection threshold—is tuned per feature to maximize their performance on the tuning dataset.

### Results and discussion

**Fig. 6:**
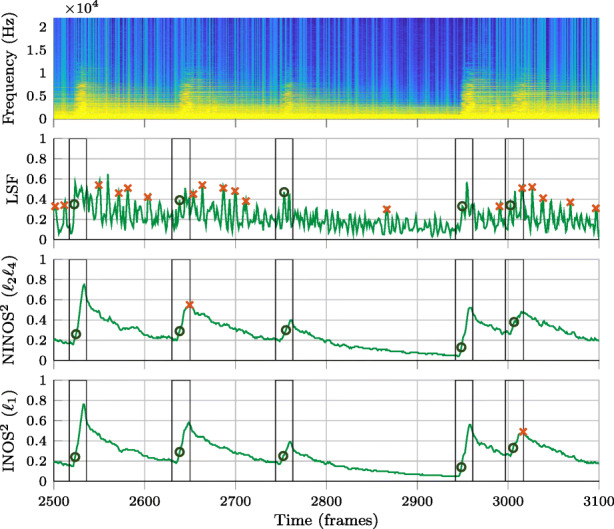
Comparison of ODFs and peak-picking results for electric guitar (major seventh stopped) excerpt. Vertical lines represent onset detection windows indicating ground-truth onsets. Circles are used to mark true positives in the peak-picking results, while false positives are marked with crosses

**Fig. 7:**
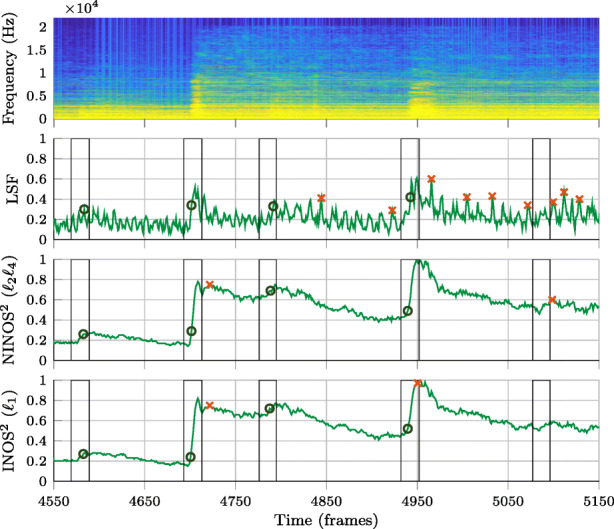
Comparison of ODFs and peak-picking results for cello (non-vibrato) excerpt. Vertical lines represent onset detection windows indicating ground-truth onsets. Circles are used to mark true positives in the peak-picking results, while false positives are marked with crosses

**Fig. 8:**
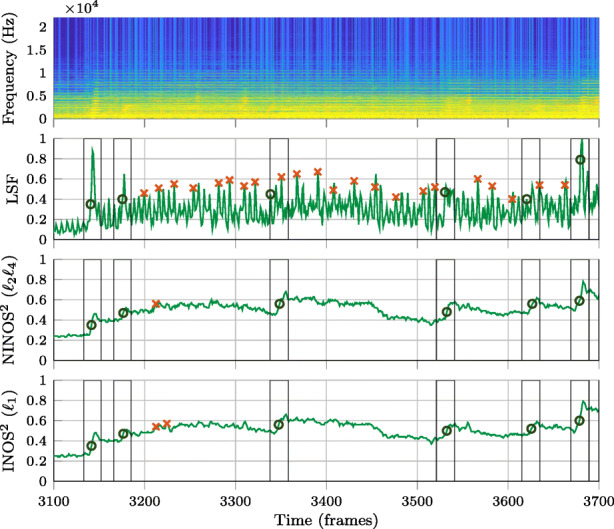
Comparison of ODFs and peak-picking results for trumpet (Bach) excerpt. Vertical lines represent onset detection windows indicating ground-truth onsets. Circles are used to mark true positives in the peak-picking results, while false positives are marked with crosses

In this section, the performance of three non-data-driven NOD methods, using the proposed NINOS^2^ (*ℓ*_2_*ℓ*_4_) ODF in (13), the proposed INOS^2^ (*ℓ*_1_) ODF in (14), and the state-of-the art LSF ODF in (5), is compared. The pre-processing introduced in Section 3.2 is only applied for the proposed methods NINOS^2^ and INOS^2^, but not to LSF. Before considering more quantitative results, we first look at a few examples of the different ODFs and the onset detections resulting from the peak picking. This is illustrated in Figs. 6–8 for three different instruments belonging to different instrument groups: the electric guitar (Fig. 6), the cello (Fig. 7), and the trumpet (Fig. 8). The examples chosen here are excerpts taken from the *P* dataset.

Onset detection windows indicating ground-truth onsets are marked with vertical black lines. While circles are used to mark true positives in the peak-picking results, false positives are marked with crosses. Finally, the false negatives are easily noted by unmarked detection windows. By visually analyzing these figures, it can be observed that the (N)INOS^2^ ODF is remarkably smoother than the LSF ODF and presents higher amplitudes at onset times for the different instruments. The lack of smoothness in the LSF ODF as compared to the (N)INOS^2^ ODF illustrates the sensitivity of the LSF ODF to nonstationary additive noise, which is partly due to the log-magnitude operation in (4). Indeed, despite the studio recording quality of the MUMS library, very-low-level nonstationary background noise can be observed in the signal spectrograms shown in Figs. 6–8, exhibiting the same temporal variation pattern seen in the LSF ODF. When moving from the easier percussive instrument in Fig. 6 to the more complicated non-percussive instruments in Figs. 7 and 8, it can be seen that the LSF ODF fails to show a clear amplitude rise at onset times. Both a high amplitude and a fast-rising amplitude at onset times are two beneficial ODF properties to have onsets successfully detected in the peak-picking step. As a consequence, the LSF ODF in Figs. 6–8 yields a higher number of false positives and false negatives after peak picking, compared to the (N)INOS^2^ ODF. A final remark that clearly stands out in the guitar example is that true positives are detected in the (N)INOS^2^ ODF on the rising edge of an ODF amplitude peak rather than at the maximum of the amplitude peak. This is a consequence of the smoothness of the ODF, resulting in an earlier onset detection which is a useful property for online NOD algorithms.

After this qualitative comparison, let us take a more quantitative approach by evaluating the previously described performance measures F1-score and detections standard deviation *σ*_*d*_. The values of these measures are shown in Tables 2, 3, 4 and 5, summarizing the results obtained for the different datasets introduced earlier (*M*, *P*, *MR8*, *PR8*). In each table, while the upper rows show the results for the different instruments organized by instrument groups, the lower rows show the results for a subset of the instruments organized into two groups depending on whether they are played with or without vibrato. The instruments included in this subset are cello, viola, violin, flute, flute alto, flute bass, and piccolo. Each table is vertically split into two parts. While the first part compares the F1-scores for the different methods, the second part compares the detections standard deviation *σ*_*d*_. Finally, the *Total* row shows the weighted average of the results for all instrument groups where the weights are determined as the relative number of instruments per group.

**Table 2:**
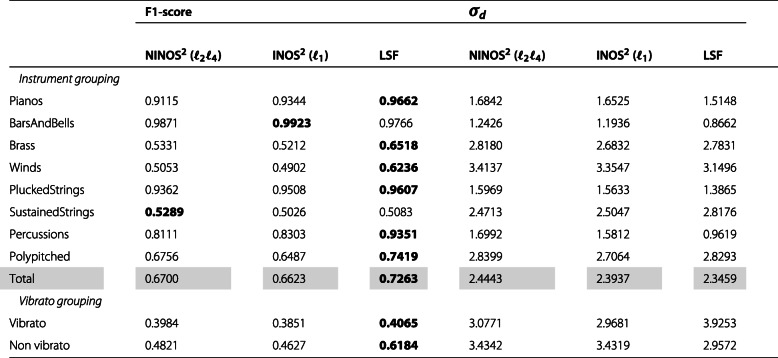
NOD performance measures for *M* dataset

**Table 3:**
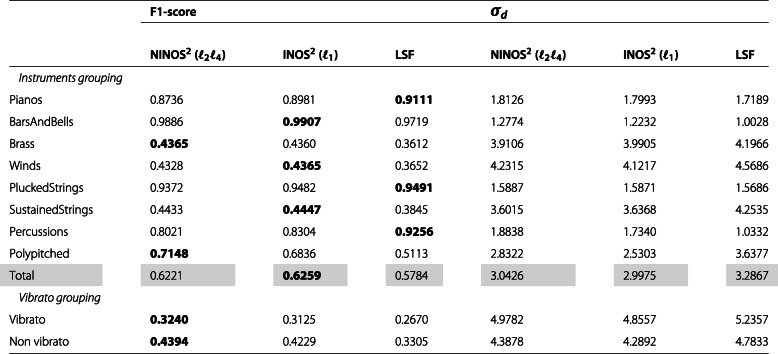
NOD performance measures for *P* dataset

**Table 4:**
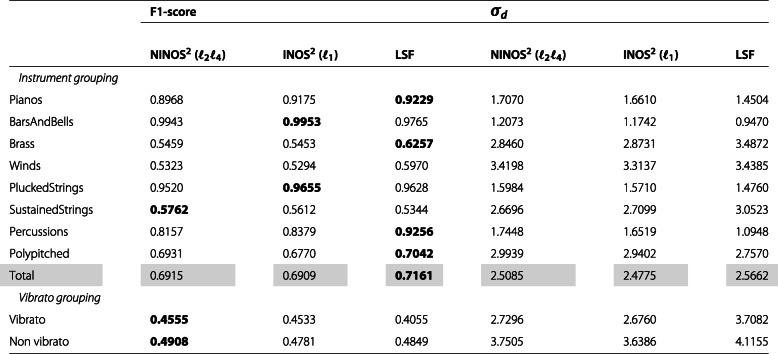
NOD performance measures for *MR8* dataset

**Table 5:**
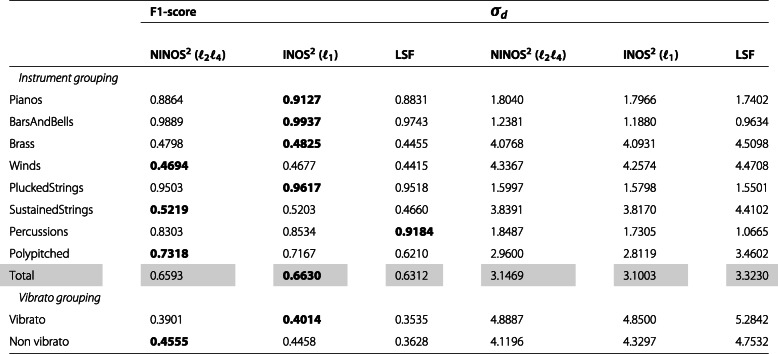
NOD performance measures for *PR8* dataset

Considering both the F1-score and the detections standard deviation *σ*_*d*_ results, a few trends can be observed. The baseline LSF ODF generally performs better on monophonic music excerpts, whereas the (N)INOS^2^ ODF is more suited for polyphonic music excerpts. Performance varies strongly across instrument groups, and for the most challenging group of sustained-strings instruments, which exhibit non-percussive and slow-attack onsets, the (N)INOS^2^ ODF outperforms the LSF ODF in all datasets. For datasets involving repeated notes, the performance gap between the different methods is slightly reduced, but the same observations can be made regarding monophonic vs. polyphonic performance for the F1-score. The detections standard deviation *σ*_*d*_ for repeated notes is however smaller with the (N)INOS^2^ ODF for both monophonic and polyphonic excerpts. For the most challenging dataset in which polyphonic excerpts with repeated notes are considered (*PR8*), the (N)INOS^2^ ODF outperforms the LSF ODF for all instrument groups except percussions. Moreover, the (N)INOS^2^ ODF seems more robust to vibrato than the LSF ODF, which can be observed in particular in the monophonic datasets (*M* and *MR8*), where the performance improvement of the (N)INOS^2^ ODF over the LSF ODF is clearly larger for the vibrato group than for the non-vibrato group. Finally, the performance of the NINOS^2^ (*ℓ*_2_*ℓ*_4_) ODF and the INOS^2^ (*ℓ*_1_) ODF is fairly similar, with a consistent trend of the NINOS^2^ (*ℓ*_2_*ℓ*_4_) ODF to perform slightly better for non-percussive instruments and the INOS^2^ (*ℓ*_1_) ODF for percussive instruments.

In order to widen the scope of the evaluation presented in this paper, the NOD performance obtained with the proposed and baseline non-data-driven ODFs is also assessed using three publicly available and differently annotated datasets, and a performance comparison with a state-of-the-art data-driven NOD method is included. The results are shown in Table 6. The MAPS CL and MAPS AM datasets are part of the MAPS^3^ dataset [33], and more specifically correspond to the music pieces portion of ENSTDkCl and ENSTDkAm. The suffixes “CL” and “AM” refer to a close-miked and ambient recording technique. Both MAPS datasets were recorded using a Yamaha Disklavier piano in which annotations are obtained directly from the instrument’s MIDI output. The MDS dataset [18] is a manually annotated dataset containing audio excerpts from various sources. It was used to train and evaluate the state-of-the-art data-driven NOD method based on a convolutional neural network (CNN) [18], which is also included in the performance comparison shown in Table 6. A 20% portion of this dataset is used for training the network and the remaining 80% is used for testing^4^. In addition, the same CNN is also tested on the two other (MAPS CL and MAPS AM) datasets. From the results in Table 6, we first observe that the variation of the F1-score across the compared methods is larger for the MDS dataset than for the two MAPS datasets. This could either be attributed to the difficulty of the NOD problem in the MDS dataset or to the higher variability of the manual annotations in the MDS dataset, or both. We also observe that while the NOD performance with the proposed (N)INOS^2^ ODF falls behind that of the other ODFs for the MDS dataset, the (N)INOS^2^ ODF outperforms both the LSF ODF and CNN for the MAPS datasets. Note that, as shown in [32], the poor performance of the CNN when evaluated on the MAPS datasets can be mitigated by aligning the training and evaluation dataset annotations by means of annotations time shifting.

**Table 6:** NOD performance measures for publicly available datasets

	NINOS^2^ (*ℓ*_2_*ℓ*_4_)	INOS^2^ (*ℓ*_1_)	LSF	CNN
MAPS_CL	0.762	**0.778**	0.728	0.759
MAPS_AM	0.458	**0.488**	0.366	0.396
MDS_TEST	0.702	0.708	0.784	**0.867**

## Conclusion and future work

In this paper, we have proposed two new variants as well as a thorough analysis of the (N)INOS^2^ spectral-sparsity-based NOD feature introduced earlier in [26]. Instead of focusing on the fundamental frequency and harmonic components of a musical note, as traditional non-data-driven NOD methods do, the (N)INOS^2^ feature uses the subset of low-energy frequency components which are found to contain valuable information on note onsets. By investigating how measures for energy and sparsity can be combined and normalized, two new spectral-sparsity-based NOD features are introduced, as well as their related ODFs describing the feature variation over time. These can be combined with a baseline peak-picking procedure to obtain a novel non-data-driven NOD method.

Simulation results for a newly developed automatically annotated semi-synthetic dataset include a large and diverse set of instruments, playing styles, and melody mixing options which are reported in terms of the F1-score and a newly introduced measure, the detections standard deviation *σ*_*d*_. In terms of the F1-score, the NOD performance using the LSF ODF is better for monophonic music excerpts, whereas the (N)INOS^2^ ODF performs better for polyphonic music excerpts. In terms of the detections standard deviation *σ*_*d*_, the benefit of the LSF ODF over the (N)INOS^2^ ODF for monophonic excerpts disappears for scenarios with repeated notes. Both performance measures illustrate that the (N)INOS^2^ ODF is more suitable than the LSF ODF for the most challenging instruments group, i.e., sustained-strings instruments, and playing style, i.e., vibrato performance.

As the proposed INOS^2^ (*ℓ*_1_) ODF is considerably cheaper to implement than the proposed NINOS^2^ (*ℓ*_2_*ℓ*_4_) ODF and the baseline LSF ODF, both in terms of computational complexity and memory usage, it seems to be the preferred feature to use for non-data-driven NOD.

Future work could include a deeper performance comparison on publicly available manually annotated NOD datasets, showing results per instrument groups. Also, in future experiments, datasets should preferably be equilibrated in terms of instruments, i.e., the different instrument groups should be populated similarly, which was not the case for the results presented in this paper. Finally, an instrument-dependent parameter tuning of the different methods and its impact on the resulting performance is worth investigating.

## Data Availability

The data that support the findings of this study are available from McGill University [35] but restrictions apply to the availability of these data, which were used under license for the current study, and so are not publicly available. Data are however available from the authors upon reasonable request and with permission of McGill University [35].

## Footnotes

1 In some publications, the SF ODF is defined by squaring the output of the half-wave rectification operator in (1) before summing over all frequency bins. Also, the lower summation index in (1) and (5) is sometimes chosen as *k*=−*N*/2 which results in a scaling of the resulting ODF with a factor of 2, due to the symmetry of the magnitude spectrum and the absence of a DC component.

2 Code and metadata available at https://gitlab.esat.kuleuven.be/dsp-public/mix-notes-mina-mounir

3 Midi Aligned Piano Sounds dataset - freely available under Creative Commons license

4 MDS excerpts partition in Train/Test folds can be retrieved from: https://gitlab.esat.kuleuven.be/dsp-public/mix-notes-mina-mounir/-/tree/master/Datasets_meta
